# Grb2 carboxyl-terminal SH3 domain can bivalently associate with two ligands, in an SH3 dependent manner

**DOI:** 10.1038/s41598-017-01364-5

**Published:** 2017-04-28

**Authors:** Richa Arya, Rohit Singh Dangi, Pinakin K. Makwana, Ambrish Kumar, Santosh Kumar Upadhyay, Monica Sundd

**Affiliations:** 1 National Institute of Immunology, Aruna Asaf Ali Marg, New Delhi, 110 067 India; 2 Department of Biochemistry, University of Delhi South Campus, Benito Juarez Road, New Delhi, 110 021 India; 3 CSIR-Institute of Genomics and Integrative Biology, Mathura Road, New Delhi, 110 020 India

## Abstract

Src homology domain containing leukocyte protein of 65 kDa (SLP65), the growth factor receptor binding protein 2 (Grb2), and the guanine nucleotide exchange factor for the Rho family GTPases (Vav), self associate in unstimulated B cells as components of the preformed B cell receptor transducer module, in an SH3-dependent manner. The complex enables the B cell to promptly respond to BCR aggregation, resulting in signal amplification. It also facilitates Vav translocation to the membrane rafts, for activation. Here we uncover the molecular mechanism by which the complex may be formed in the B cell. The C-terminal SH3 domain (SH3C) of Grb2 bivalently interacts with the atypical non-PxxP proline rich region of SLP65, and the N-terminal SH3 domain (SH3N) of Vav, both the interactions crucial for the proper functioning of the B cell. Most surprisingly, the two ligands bind the same ligand binding site on the surface of Grb2 SH3C. Addition of SLP65 peptide to the Grb2-Vav complex abrogates the interaction completely, displacing Vav. However, the addition of Vav SH3N to the SLP65-Grb2 binary complex, results in a trimeric complex. Extrapolating these results to the *in vivo* conditions, Grb2 should bind the SLP65 transducer module first, and then Vav should associate.

## Introduction

Stimulation-independent SLP65 assemblies have been observed in the resting B cell cytosol, necessary for the membrane translocation of SLP65, its Syk mediated phosphorylation, and subsequent activation of Ca^2+^ and NF-κB responses upon BCR stimulation^1^. Genetic reconstitution experiments demonstrate the requirement of these preformed complexes in the initiation of B cell signaling^1^, delivering the preassembled SLP65 signaling cargo to the activated BCR, resulting in signal amplification^1–4^. The assemblies comprise the Src homology domain containing leukocyte protein of 65 kDa (SLP65), the Cbl-protein of 85 kDa (CIN85)^4^, and several other signaling proteins.

Within this preformed B cell receptor transducer module, SLP65 constitutively associates with several signaling proteins, *viz*. the Growth factor receptor binding protein-2 (Grb2), which in turn binds the nucleotide exchange factor Vav. This unique trimeric complex enables the B cell to respond to BCR aggregation without any delay^1, 5^. The first evidence for the existence of this complex came from co-immunoprecipitation studies, when the anti-SLP65 antibodies precipitated Grb2 and Vav-1 in unstimulated and pervanadate/H_2_O_2_ stimulated J5888Lμm3 cells. The phosphorylated form of SLP65 was observed only in the lysates of pervanadate/H_2_O_2_-stimulated cells, suggesting constitutive association of SLP65, Grb2, and Vav in unstimulated cells^6^. Since then, Grb2 has been identified in the proteome^6–8^ and interactome^1^ of SLP65 in the resting B cells, while Grb2 and Vav were present together as early interactors of SLP65 post BCR stimulation^1^. Grb2 has been recognized as a component of the CIN85 complex, in association with unphosphorylated SLP65 and Vav^9, 10^. Likewise, Vav-1 and SLP65 have been identified in the Grb2 interactome, in the activated B cells^11^. Vav-1 and Vav-3, both associate with Grb2 and SLP65 *in vivo*, in an SH3-dependent manner^6, 12^. The association translocates Vav to the lipid rafts, where it is activated^6, 13–15^.

SLP65^16^ and Grb2^17^ are multidomain, enzymatically inert adaptor proteins, with manifold functions. SLP65 comprises an N-terminal basic region, a proline rich region, and a C-terminal SH2 domain. It displays high sequence similarity to SLP76, its paralog in the T cells^6^. Grb2 is comparatively smaller, containing two SH3 domains, flanking a central SH2 domain, connected by a flexible linker^18^. *In vivo*, full length Grb2 exists in a monomer-dimer equilibrium, and their ratio determines normal/oncogenic function^19^. The dimer is stabilized by a salt bridge between Y160 present in the C-terminal SH3 domain of Grb2, and E87 residing in the SH2 domain of the other protomer. The equilibrium shifts to the monomer form upon either phosphorylation of Y160 of Grb2, binding to a phosphorylated ligand, or mutation of Y160 to a glutamate^19^. Vav belongs to a large family of guanine nucleotide exchange factors for the Rho family GTPases. Vav-1, Vav-2 and Vav-3 are structurally similar multi-domain proteins, >50% identical, with overlapping functions^12, 20^. The decrease in BCR-induced calcium mobilization in Vav-3 deficient DT40 B cells could be rescued by overexpression of Vav-2^21^. All three Vav members activate Rho GTPases *in vitro* ^22^.

To date, only a part of the SLP65-Grb2-Vav trimeric complex has been structurally characterized *i.e*. the Grb2-Vav binary complex^13, 14^. A similar interaction between the Vav SH3N domain and the Nck SH3C domain has also been observed^23^. Though no information exists for the Grb2-SLP65 interaction, structural information is available for an analogous interaction, *i.e*. the SH3C Mona/Gads-SLP76 interaction^11, 12, 24, 25^. Here, for the first time, we uncover the molecular mechanism by which a stimulation independent trimeric complex forms between Grb2, SLP65 and Vav in the resting B cell. Our studies bring to light, a unique aspect of the Grb2 SH3C domain that has been overlooked so far, *i.e*. its ability to associate with two ligands, simultaneously.

## Results

Biochemical studies underscore the importance of a unique SH3-dependent SLP65-Grb2-Vav trimeric complex in B cell function^2, 6, 26^. Grb2-Vav interaction has been shown to occur by means of the carboxyl-SH3 domain (SH3C) of Grb2 and the tetra-proline sequence present in the RT loop of Vav N-terminal SH3 (SH3N) domain^13, 14^. Similarly, the SLP65 sequence 204 PMVNRSTKP 212 present in the proline rich region, associates with the ligand binding site of Mona/Gads SH3C domain, a relative of Grb2^27^. Thus, to characterize the SLP65-Grb2-Vav trimeric complex using NMR, the SH3C domain of Grb2, the SH3N domain of Vav1 and the proline rich region of SLP65 (residues 125–330) were used as an alternative for full length proteins. The purity of the purified proteins on SDS PAGE and Native PAGE gel is shown in Supplementary Fig. S1a,b. Glutaraldehyde crosslinking studies and size exclusion chromatography confirmed the monomeric states of Grb2 SH3C and Vav SH3N domains (Supplementary Fig. S1c,d).

The binary complexes *viz*. SLP65-Grb2, and Grb2-Vav were generated first, using ^15^N^13^C labeled Grb2 SH3C domain and unlabeled SLP65/Vav, and characterized using NMR. Unlabeled Vav and SLP65 domains were subsequently added to the two complexes respectively, to form the SLP65-Grb2-Vav trimeric complex.

### Grb2 SH3C interacts with the atypical proline rich region of SLP65

**Figure 1:**
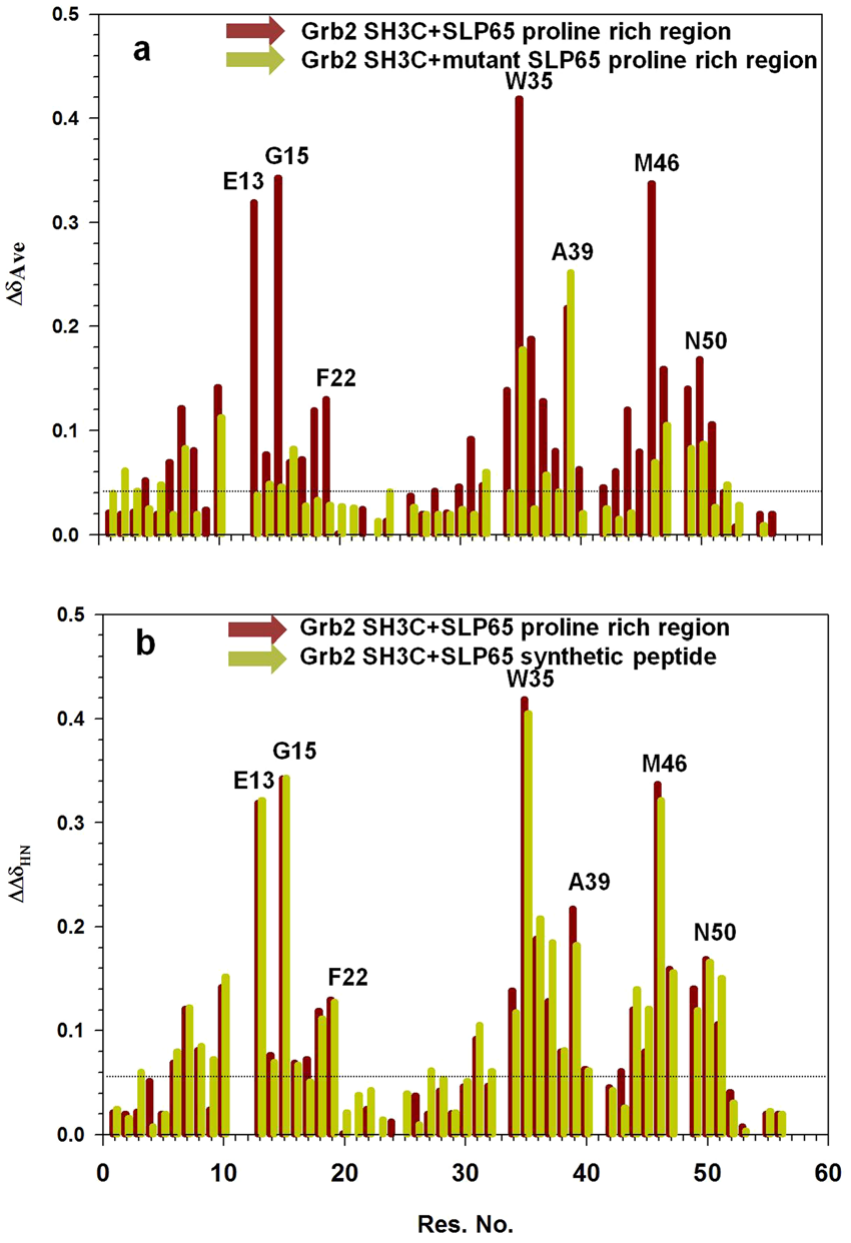
Backbone amide chemical shift perturbations of the Grb2 SH3C domain upon binding SLP65. Comparison of the average chemical shift changes of the Grb2 SH3C amides upon addition of (**a**) wild type SLP65 proline rich region (residues 125–330, brown) and P212A mutant of the proline rich region (green). (**b**) the wild type proline rich region of SLP65 (brown) and a synthetic peptide Ace-EKAPMVNRSTKPNSSS-NH2 (green).

Immunoprecipitation studies in mice have shown that Grb2 SH3C interacts with SLP65 *in vivo*. To understand the interaction at the molecular level, a ^15^N^13^C labeled sample of Grb2 SH3C was titrated with unlabeled proline rich region of SLP65 (residues 125–330, UniProt Q9QUN3). Noticeable changes in chemical shift were observed for several backbone amides, (Fig. 1a, brown bars), consistent with ligand binding.

In order to confirm that 204 PMVNRSTKP 212 sequence of mice SLP65 is indeed the region that binds Grb2 SH3C domain, site directed mutagenesis studies were carried out. The proline residue following the lysine in the aforementioned sequence (residues 125–330) was mutated to an Ala (P212A), and its binding with ^15^N labeled Grb2 SH3C was followed using NMR. A remarkable decrease in the amplitude of chemical shift perturbations was observed for several Grb2 SH3C amides upon binding the P212A SLP65 mutant (green bars), as compared to the wild type protein (brown) (Fig. 1a). In SLP76 studies, the mutation of the same proline in the sequence ‘P-X_3_-R-X_2_-K-P’ to a valine causes ~7-fold reduction in its affinity for Mona/Gads SH3C^27, 28^.

The proline rich region of SLP65 was unstable at room temperature when left for long periods. Therefore, a synthetic peptide corresponding to the sequence 201–216 of mice SLP65 (Ace-EKAPMVNRSTKPNSSS-NH2) was synthesized. The binding of Grb2 SH3C to this peptide was compared to its binding with SLP65 proline rich region (residues 125–330). Figure 1b displays the chemical shift perturbations of the backbone amides of Grb2 SH3C upon binding the proline rich region of SLP65 (brown bars), superimposed on the perturbations observed upon binding the synthetic peptide (green bars). The perturbations observed in the two binding studies were of equal magnitude, suggesting equivalent binding. The synthetic peptide was therefore used as a substitute for the proline rich region of SLP65 in the rest of the studies.

**Figure 2:**
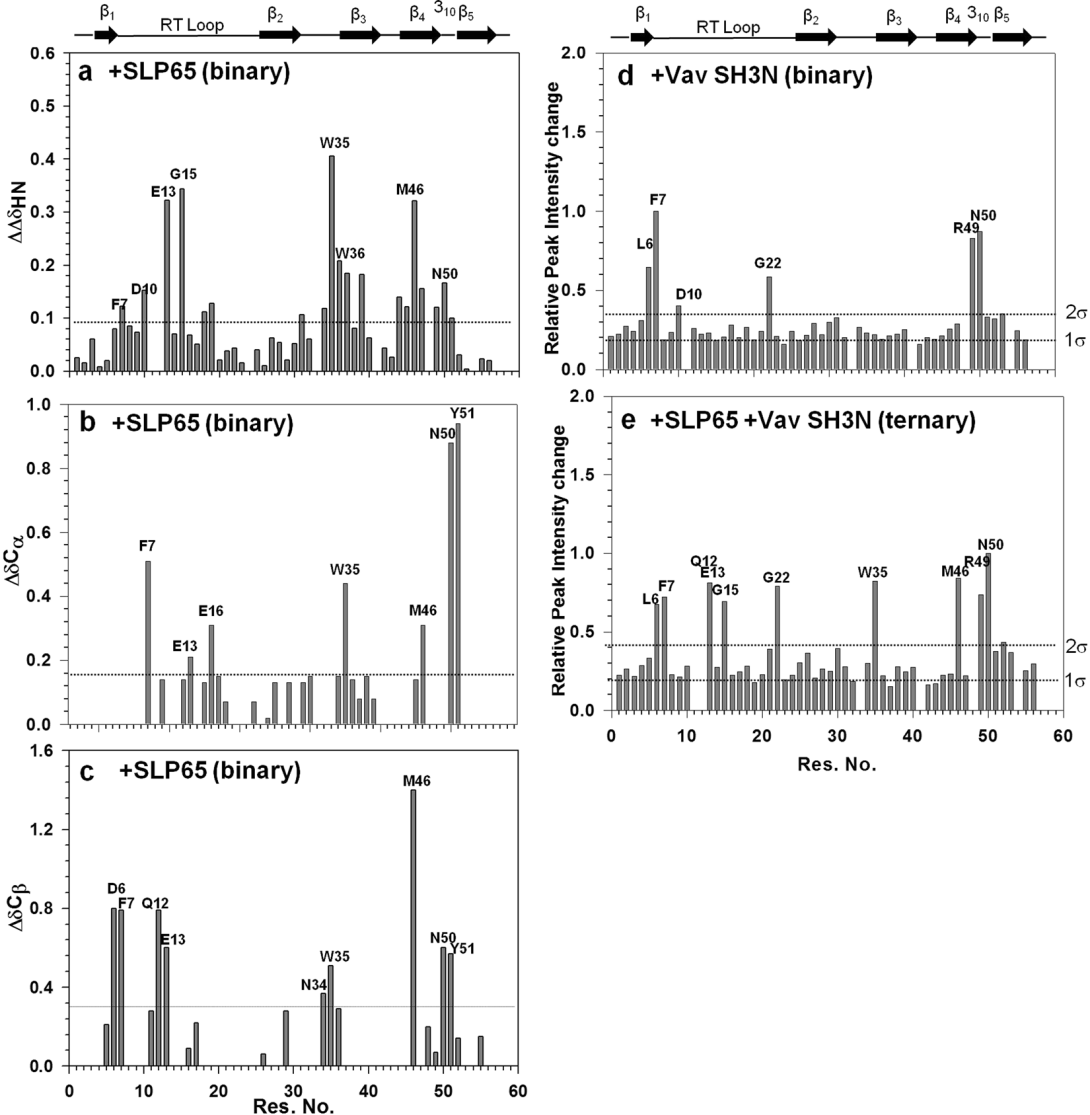
The interaction of Grb2 SH3C domain with its ligands. Changes in the (**a**) Average amide, (**b**) C_α_, and (**c**) C_β_ chemical shifts of Grb2 SH3C domain upon binding the SLP65 peptide. Changes in the (**d**) peak intensities of the Grb2 SH3C amides upon binding Vav SH3N domain in the binary complex, (**e**) peak intensities of Grb2 SH3C amides upon binding Vav SH3N domain in the trimeric complex. The binding studies were carried out in 20 mM Sodium Phosphate buffer, 100 mM NaCl, pH 6.0, 25 °C. A discontinuous horizontal line in the figure marks one or two standard deviations.

**Figure 3:**
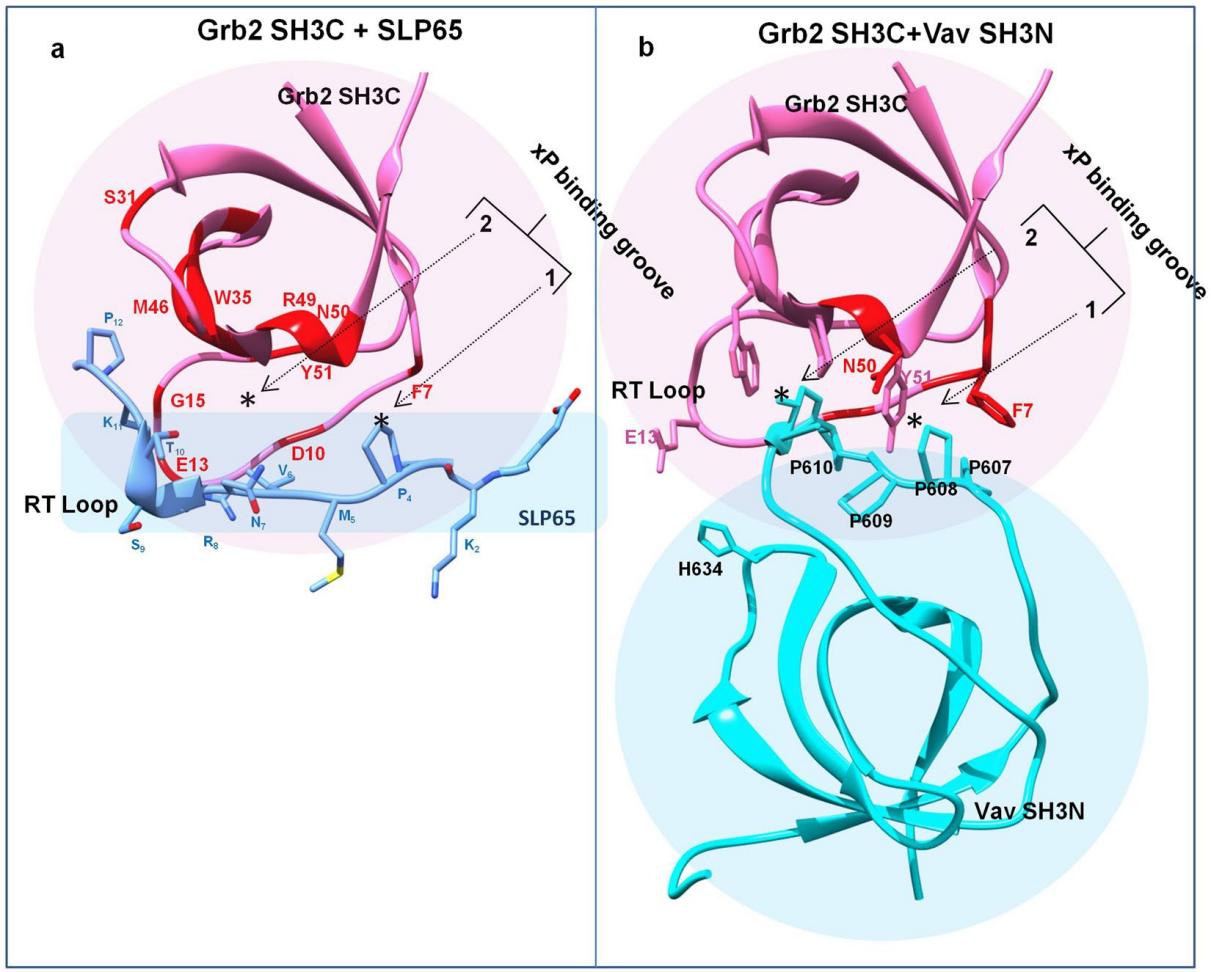
Comparison of the interaction interface of the Grb2 SH3C complexes. A ribbon representation of the (**a**) Grb2 SH3C in complex with SLP65 peptide (based on 2VWF), and (**b**) Grb2 SH3C domain in complex with Vav SH3N domain (1GCQ). The backbone amides that display chemical shift changes in the Grb2 SH3C domain in our studies are highlighted in red.

A close examination of the ^1^H^15^N Grb2 SH3C-SLP65 peptide interaction (1:6 final molar ratio) suggests notable chemical shift perturbations of several backbone amides of Grb2 SH3C. Supplementary Fig. S2a shows the ^1^H^15^N HSQC spectra for free Grb2 SH3C, overlaid on its spectra with increasing SLP65 peptide concentrations (red 0; pink 0.4; green 1.4; blue 2fold molar excess). Some of the amides that display large chemical shift changes have been highlighted and labeled in the figure. The amides of Phe 7, Asp 10, Glu 13, Gly 15, Gly 18, Phe 19, Trp 35, Trp 36, Lys 37, Ala 39, Thr 44, Gly 45, Met 46, Phe 47, Arg 49, Asn 50 and Tyr 51 displayed changes greater than one standard deviation, as illustrated (Fig. 2a). These amides were mapped (color red) on the structure of Grb2 SH3C-SLP65 peptide complex as illustrated in Fig. 3a (using Grb2-Gab2 peptide complex PDB 2VWF, and mutating Gab2 peptide to the SLP65 sequence).

Changes in C_α_ chemical shift were also followed, as they primarily report alteration in the dihedral angles^29, 30^. Phenylalanine 7, Glu 13, Glu 16, Trp 35, Met 46, Asn 50 and Tyr 51 displayed significant C_α_ change (Fig. 2b). C_β_ chemical shift changes were observed for Asp 6, Phe 7, Gln 12, Glu 13, Asn 34, Trp 35, Met 46, Asn 50, and Tyr 51 (Fig. 2c). Three side chains also displayed noticeable changes in chemical shift upon peptide interaction, *i.e*. ND2 & HD21/HD22 of Asn 34, HE1 of Trp 35, and ND2 of Asn 50.

### Grb2 SH3C forms a binary complex with the SH3N of Vav

Biochemical and structural studies have shown that Grb2 SH3C also forms a complex with the SH3N of Vav. Vav SH3N is structurally similar to other SH3 domains (all beta sheet conformation), with the exception that it does not bind proline rich peptides *via* its ligand binding surface, as its ligand binding site is blocked by its own RT loop.

To understand the formation of the Grb2-Vav binary complex, a ^15^N^13^C labeled Grb2 SH3C sample was titrated with unlabeled Vav SH3N (1:2 final molar ratio). Line broadening greater than average was observed for several backbone amides, *i.e*. Leu 6, Phe 7, Asp 10, Gly 22, Arg 49 and Asn 50 as shown in Supplementary Fig. S2b. Relative changes in peak intensities of the Grb2 SH3C amides upon Vav binding have been plotted as a function of residue number in Fig. 2d. Two standard deviations were used as cutoff. To get an in-depth picture of the interaction, the Grb2 SH3C amides that display line broadening upon Vav interaction in our studies have been mapped (color red) on the X-ray structure of the Grb2 SH3C (pink)-Vav SH3N complex (cyan) (PDB 1GCQ) in Fig. 3b.

### SLP65 displaces Vav SH3N from the Grb2-Vav complex, giving rise to Grb2-SLP65 complex

As the order in which the three proteins associate *in vivo* to form the trimeric complex (Vav-Grb2-SLP65) remains unknown, the third binding partner was added to the two binary complexes, *i.e*. SLP65 peptide was added to the Grb2-Vav complex (resulting in Complex 1), and in a separate experiment, Vav was added to the Grb2-SLP65 complex (giving rise to Complex 2). A final Grb2:SLP65 molar ratio of 1:6 was attained in Complex 1, and 1:6 Grb2:Vav final molar ratio in Complex 2. Changes in the amide chemical shift, a hallmark of fast time scale exchange, was used as a probe for SLP65 peptide binding, while anomalous line broadening was used as an indicator of Vav SH3N binding.

**Figure 4:**
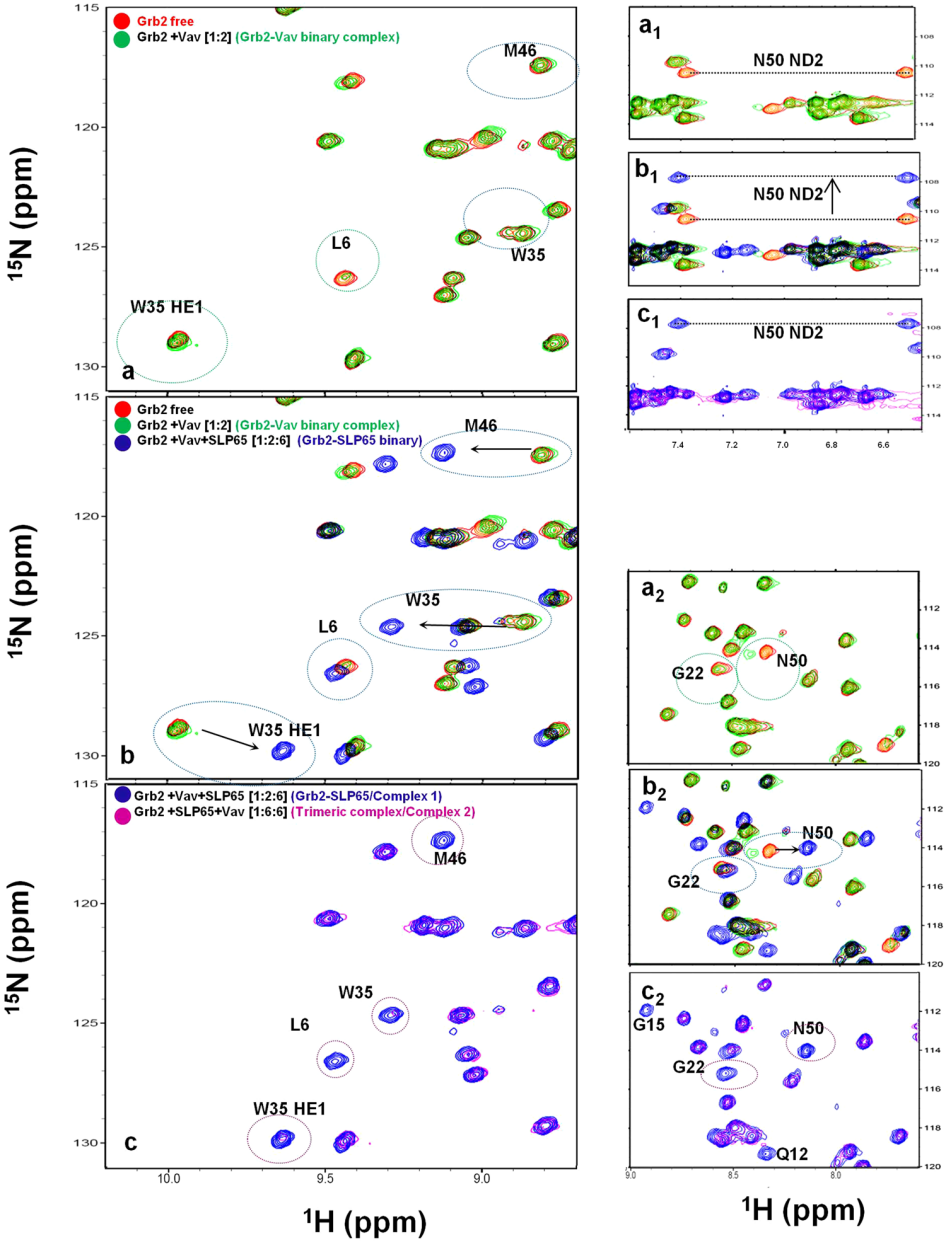
^1^H^15^N HSQC spectra of the Grb2 SH3C domain in complex with its ligands. (**a**,**a**_**1**_,**a**_**2**_) ^1^H^15^N HSQC spectra of the free Grb2 SH3C (red), superimposed on the spectra of Grb2SH3C-Vav SH3N complex, 1:2 molar ratio (green). (**b**,**b**_**1**_,**b**_**2**_) Multiple overlaid spectra of free Grb2 SH3C (red), Grb2 SH3C-Vav SH3N complex (green), and Grb2 SH3C-Vav SH3N complex after addition of six-fold molar excess of SLP65 peptide, resulting in Grb2-SLP65 complex (Complex 1, blue). (**c**,**c**_**1**_,**c**_**2**_) Overlaid spectra of the Grb2-SLP65 complex, (Complex 1, blue), and the trimeric complex SLP65-Grb2-Vav, formed upon addition of six-fold excess of Vav SH3N (Complex 2, magenta).

Figure 4a,a_1_,a_2_ display regions of the ^1^H^15^N HSQC spectra for free Grb2 SH3C (colored red), superimposed on the spectra of its complex with Vav SH3N (colored green). In the figure, the peaks for Leu 6, Gly 22 and Asn 50 of Grb2 SH3C show line broadening in the Grb2-Vav complex (colored green). Upon addition of SLP65 to this sample (resulting in Complex 1), a discernible increase in the peak intensity of the Grb2 SH3C amides was observed (amides that were broadened in the Grb2-Vav binary complex), suggesting the release of Vav from the complex. A concomitant change in the chemical shift of the Grb2 SH3C amides was also observed, analogous to SLP65 peptide binding. As shown in Fig. 4b,b_1_,b_2_, Leu 6, Gly 22 and Asn 50 amides broadened in the Grb2-Vav complex (green) get intense upon formation of Complex 1 (color blue). The side chain ND2 of Asn 50, that disappears in the Grb2-Vav complex due to line broadening, also reappears in complex 1 (colored blue, Fig. 4c_1_), at a lower nitrogen chemical shift value, in compliance with the SLP65 bound state. These results suggest that the addition of SLP65 to the Grb2-Vav binary complex, gives rise to Grb2-SLP65 binary complex (Complex 1).

### Addition of Vav SH3N to the Grb2-SLP65 binary complex, results in a trimeric/ternary complex

The addition of Vav SH3N to the Grb2-SLP65 binary complex (to form Complex 2), did not release SLP65 peptide from the complex, as disclosed from the unaltered amide chemical shifts. However, severe line broadening was observed for several Grb2 SH3C amides, in agreement with Vav SH3N binding. Nearly all the Grb2 SH3C amides (except Asp 10) that display line broadening in the Grb2-Vav binary complex, also display broadening in Complex 2, at a chemical shift value consistent with the SLP65 bound conformation of Grb2 SH3C, as illustrated in Figs 2e and 4c. Line broadening observed in the Grb2 SH3C amides upon Vav binding in the Grb2-Vav binary complex and SLP65-Grb2-Vav trimeric complex (Complex 2) has been compared in Supplementary Fig. S3. Notably, several more amides near the xP binding groove 2, *i.e*. Gln 12, Glu 13, Gly 15, Trp 35 and Met 46, display broadening of line widths in Complex 2.

**Figure 5:**
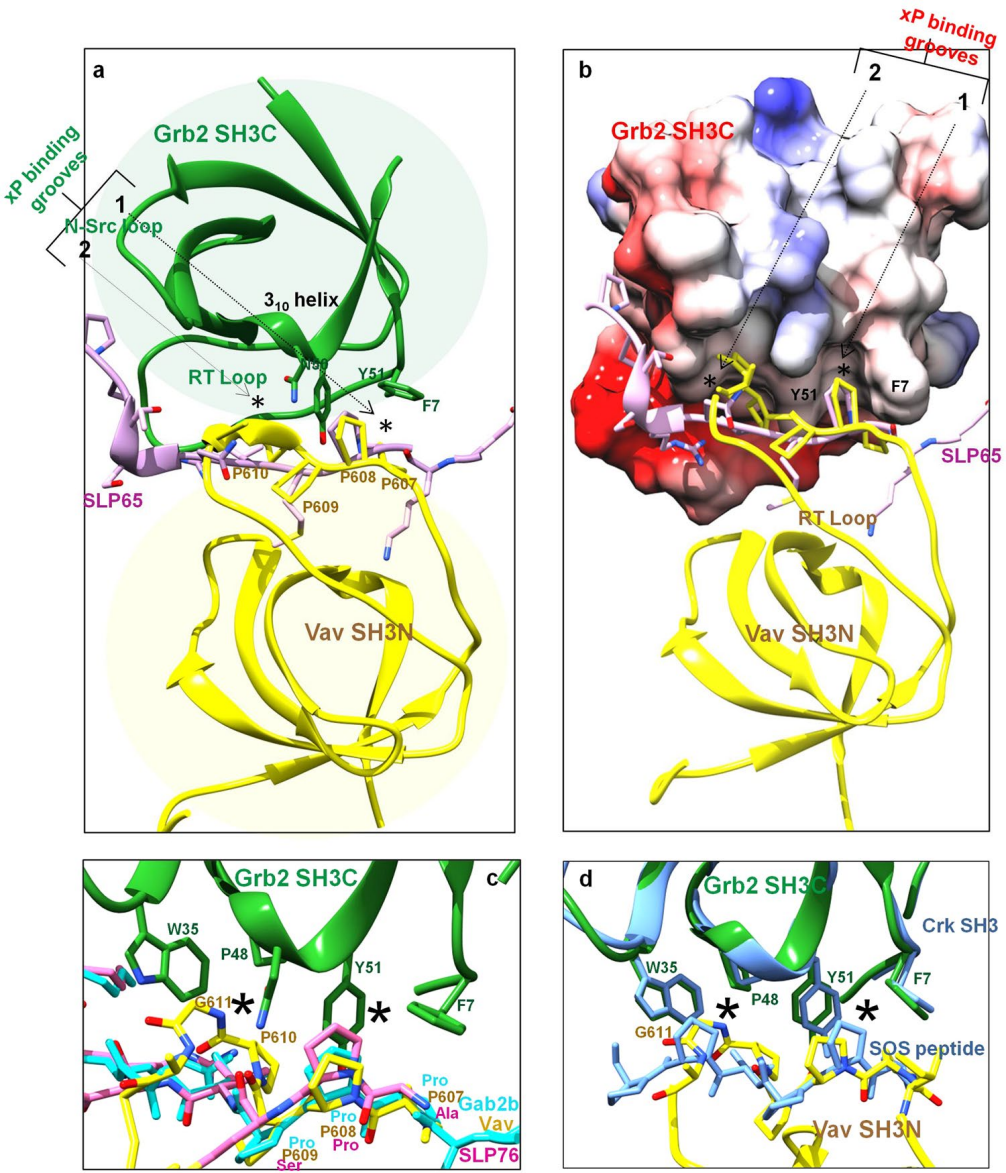
A proposed model for the SLP65-Grb2-Vav trimeric complex. (**a**) A model of the trimeric complex of Grb2 SH3C domain (green) with the SLP65 peptide (Ace-EKAPMVNRSTKPNSSS-NH2), colored pink, and Vav SH3N domain (yellow) shown as ribbons. The model was prepared by superimposing the structures 1GCQ and 2VWF, using Chimera^31^. The side chains of the Grb2 SH3C domain that interact with Vav are shown in sticks. (**b**) The trimeric complex, displaying Grb2 SH3 as surface. (**c**) The interaction interface of Grb2 SH3C/Mona Gads with the ligands Vav (yellow), Gab2b (pink) and SLP76 (cyan) in the crystal structures 1GCQ, 2VWF, and 1OEB, respectively. (**d**) Interaction interface of Grb2 SH3C-Vav SH3N overlaid on the Crk SH3C-SOS peptide complex.

Comparing the amide peaks in Complex 1 (colored blue) and Complex 2 (colored magenta) as shown in Fig. 4c,c_1_,c_2_, it is evident that the Complex 2 is a trimeric complex, formed by the addition of Vav SH3N to the Grb2-SLP65 complex. Combining these chemical shift perturbation/line broadening studies, with the available structural data (1GCQ: Grb2 SH3C-Vav SH3N complex and 2VWF: Grb2 SH3C-Gab2 peptide complex), we propose a model for the structure of the trimeric complex of Grb2-SLP65-Vav, as illustrated in Fig. 5a,b. The figure has been generated by overlaying the two aforementioned structures, using the matchmaker option of Chimera, and mutating the residues of Gab2 peptide to match the SLP65 sequence^31^. Figure 5a displays the modeled SLP65-Grb2-Vav trimeric complex with Grb2 SH3C (green ribbons), Vav SH3N (yellow ribbons) and SLP65 peptide (pink ribbons), while in Fig. 5b, the same complex is shown with Grb2 SH3C in surface representation. The xP binding grooves are shown as asterisk in the figure.

Figure 5c displays the interaction interface of Grb2 SH3C (green) with Vav SH3N containing ‘PPPPG’ sequence (yellow, PDB 1GCQ), Gab2b peptide ‘QPPPVNRNLKPDRKAKPT’ (pink, PDB 2VWF), and SLP76 peptide ‘PAPSIDRSTKPP’ (cyan, 1OEB, Mona/Gads -SLP76 peptide complex). The proline in Gab2b, corresponding to Pro 608 of Vav, structurally overlaps with the latter in the crystal structure of the complex, while the corresponding proline in the SLP76 peptide lies slightly above it in its complex with Mona/Gads SH3C domain. Though we have modeled the SLP65 peptide in the trimeric complex using PDB 2VWF as a template, the proline may attain a conformation similar to SLP76 peptide in its complex with Mona/Gads SH3C domain, as they share a consensus sequence ‘APxxNRSTKP’, lacking the PPP sequence, conserved in Vav and Gab2b.

Figure 5d shows the interaction interface of Grb2 SH3C-Vav SH3N complex (PDB 1GCQ) superimposed on the Crk SH3-SOS peptide PPPVPPRRR complex (PDB 1CKB). Notably, the polyproline helix backbone of Vav SH3N (yellow), formed by the PPPPG sequence is very similar to the SOS peptide canonical polyproline helix formed by PPVPP sequence (sky blue) (Fig. 5d), and remarkably different from the SLP65 peptide that lacks the polyproline helix (Fig. 5c).

### Vav SH3N does not directly interact with the proline rich region of SLP65

To verify whether SLP65 at all interacts with Vav SH3N in the SLP65-Grb2-Vav trimeric complex, ^15^N^13^C labeled Vav SH3N was titrated with unlabelled SLP65 peptide (1:4 molar ratio). Insignificant changes in chemical shift were observed (data not shown), ruling out the possibility of any direct interaction between the two.

### SLP65 modulates the affinity of Grb2 SH3C for Vav SH3N

**Figure 6:**
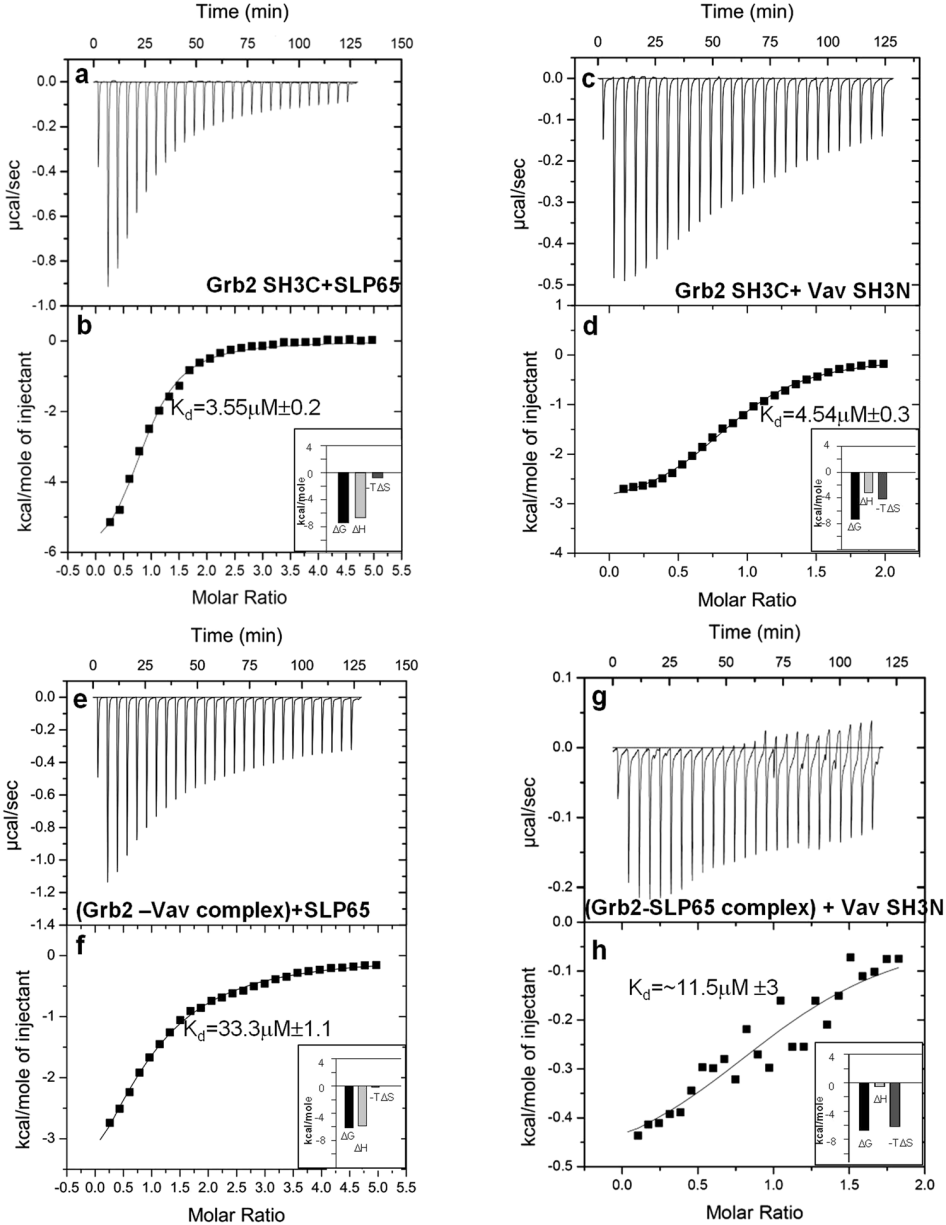
Isothermal Titration Calorimetry measurements. ITC titration profiles for the free Grb2 SH3C domain upon binding (**a** and **b**) the SLP-65 peptide, (**c** and **d**) Vav SH3N domain. The titration of (**e** and **f**) the Grb2-Vav complex with SLP65 peptide (resulting in Complex 1), and (**g** and **h**) Grb2-SLP65 with Vav SH3N (giving rise to Complex 2). The upper panels in the figure represent the ITC thermograms displaying the time dependent deflection of heat after each injection, and the lower ones display peak integrals as a function of molar ratio.

**Figure 7:**
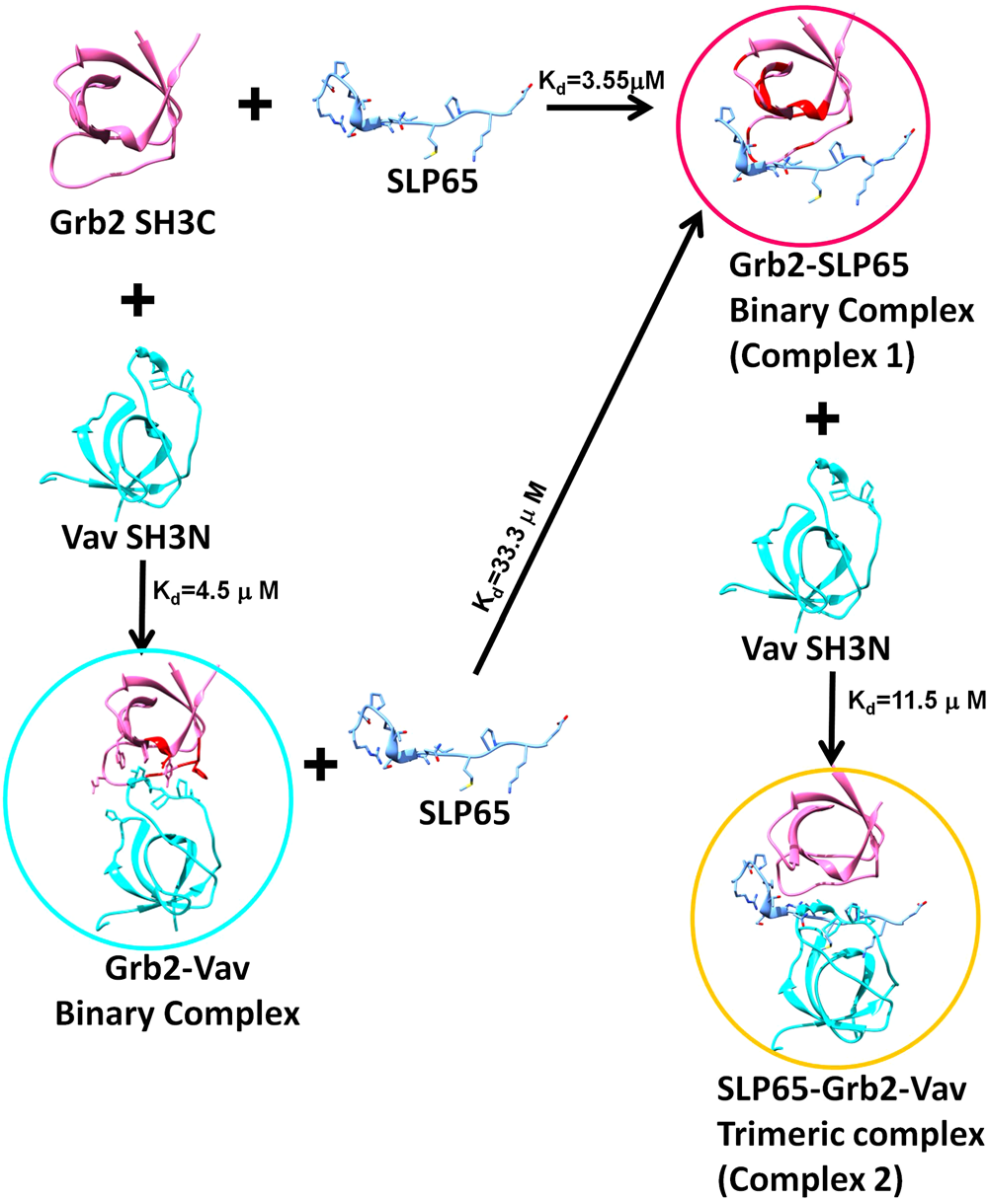
Schematic diagram depicting the formation of the binary and trimeric complexes. Schematic representation of the monovalent and bivalent modes of interaction of Grb2 SH3C with its ligands SLP65 and Vav. Binding of SLP65 or Vav to Grb2 SH3C results in a binary complex, Grb2-SLP65 or Grb2-Vav, respectively. Addition of SLP65 to the Grb2-Vav binary complex results in Grb2-SLP65 binary complex (Complex 1). However, the addition of Vav SH3N to the Grb2-SLP65 binary complex, gives rise to a trimeric complex (Complex 2).

The binding of Grb2 SH3C with the two ligands was also followed by ITC measurements, that provide a thermodynamic finger print of the binding process. Grb2 SH3C was titrated with SLP65 peptide in one experiment, and Vav SH3N in the other. A K_d_ value of 3.55 μM was obtained for SLP65 binding, while 4.5 μM was observed for Vav SH3N binding. The binding isotherms for the two independent sets of experiments are illustrated in Fig. 6a–d. The data were fitted to a one-site binding model, and the thermodynamic parameters for the two binding events are listed in Table 1. ITC measurements were also carried out on the two binary complexes, *i.e*. Grb2-Vav and Grb2-SLP65. SLP65 peptide was added to the Grb2-Vav complex to form Complex 1. A K_d_ value of 33.3 μM was obtained, (Fig. 6e,f and Table 1). Similarly, the titration of the SLP65-Grb2 binary complex with Vav SH3N to form Complex 2 resulted in a decrease in the binding enthalpy, increase in entropy and K_d_ as shown in Fig. 6g,h. Only an approximate K_d_ (~11.5 μM) value could be obtained. Based on the NMR and ITC results, a schematic representation of the binding modes of Grb2 SH3C with its ligands SLP65 or Vav, is illustrated in Fig. 7.

**Table 1:** Thermodynamic parameters obtained using ITC for the binding of Grb2 SH3C with the SLP65 peptide and Vav SH3N.

Ligand	K_d_ (μM)	n	ΔH (kcal/mol)	−TΔS (kcal/mol)	ΔG (kcal/mol)
SLP65 peptide	3.55 ± 0.2	0.87 ± 0.02	−6.67 ± 0.15	−0.76 ± 0.1	−7.43 ± 0.03
SLP65 peptide^a^	33.3 ± 1.1	0.91 ± 0.01	−5.9 ± 0.04	−0.2 ± 0.03	−6.1 ± 0.02
Vav SH3N	4.5 ± 0.3	0.91 ± 0.05	−3.15 ± 0.04	−4.14 ± 0.09	−7.29 ± 0.02
Vav SH3N^b^	11.5 ± 3	1.10 ± 0.1	−0.54 ± 0.08	−6.19 ± 0.04	−6.73 ± 0.05

^a^The ligand was added to the Grb2-Vav binary complex, giving rise to Complex 1. ^b^The ligand was added to the Grb2-SLP65 peptide complex, giving rise to Complex 2 (trimeric complex).

### Line broadening of the Grb2 SH3C amides in its complex with Vav SH3N is a consequence of conformational exchange

**Figure 8:**
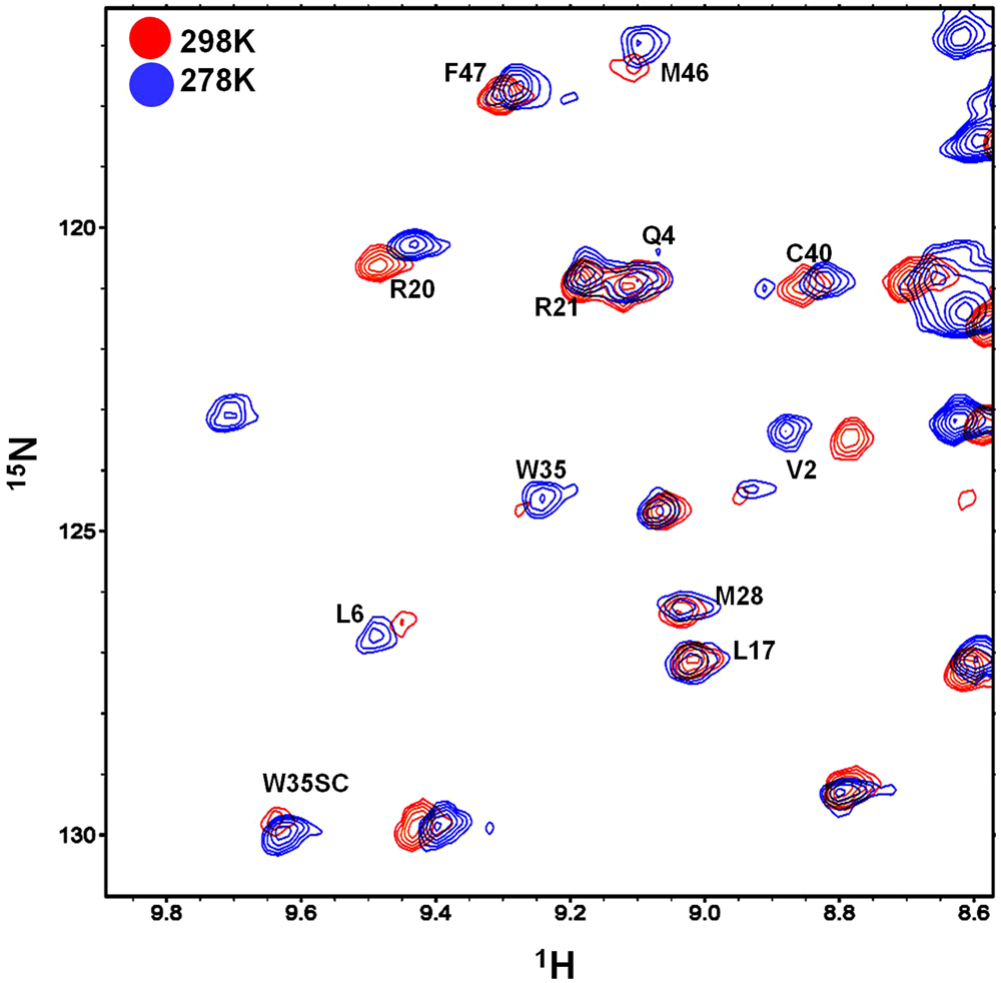
Temperature dependence of the backbone amides. Spectra displaying peak intensities of some of the backbone amides of the Grb2 SH3C domain in the SLP65-Grb2-Vav trimeric complex at room temperature (colored red), overlaid on the spectra acquired at 10 °C (colored blue).

Several amides of the Grb2 SH3C domain display exchange broadening upon interaction with Vav SH3N in the ^1^H^15^N HSQC spectra. Peaks that display line broadening in the trimeric complex at 298 K *viz*. Leu 6, Gly 22, Trp 35 (including side chain), Met 46 and Asn 50 (including side chain), became intense upon lowering the temperature to 283 K, as shown in Fig. 8. Conceivably, the chemical exchange process occurring in the intermediate exchange regime at 298 K, shifts to the slow exchange regime at 283 K.

### Binding of Vav SH3N induces slow dynamics in the Grb2 SH3C backbone

The dynamics of Grb2 SH3C in the a) free form, b) binary complex with the SLP65 peptide and c) the trimeric complex with SLP65 peptide and Vav SH3N, were followed by ^15^N relaxation dispersion experiments, which provide kinetic as well as thermodynamic information regarding the μs-ms exchange process.

**Figure 9:**
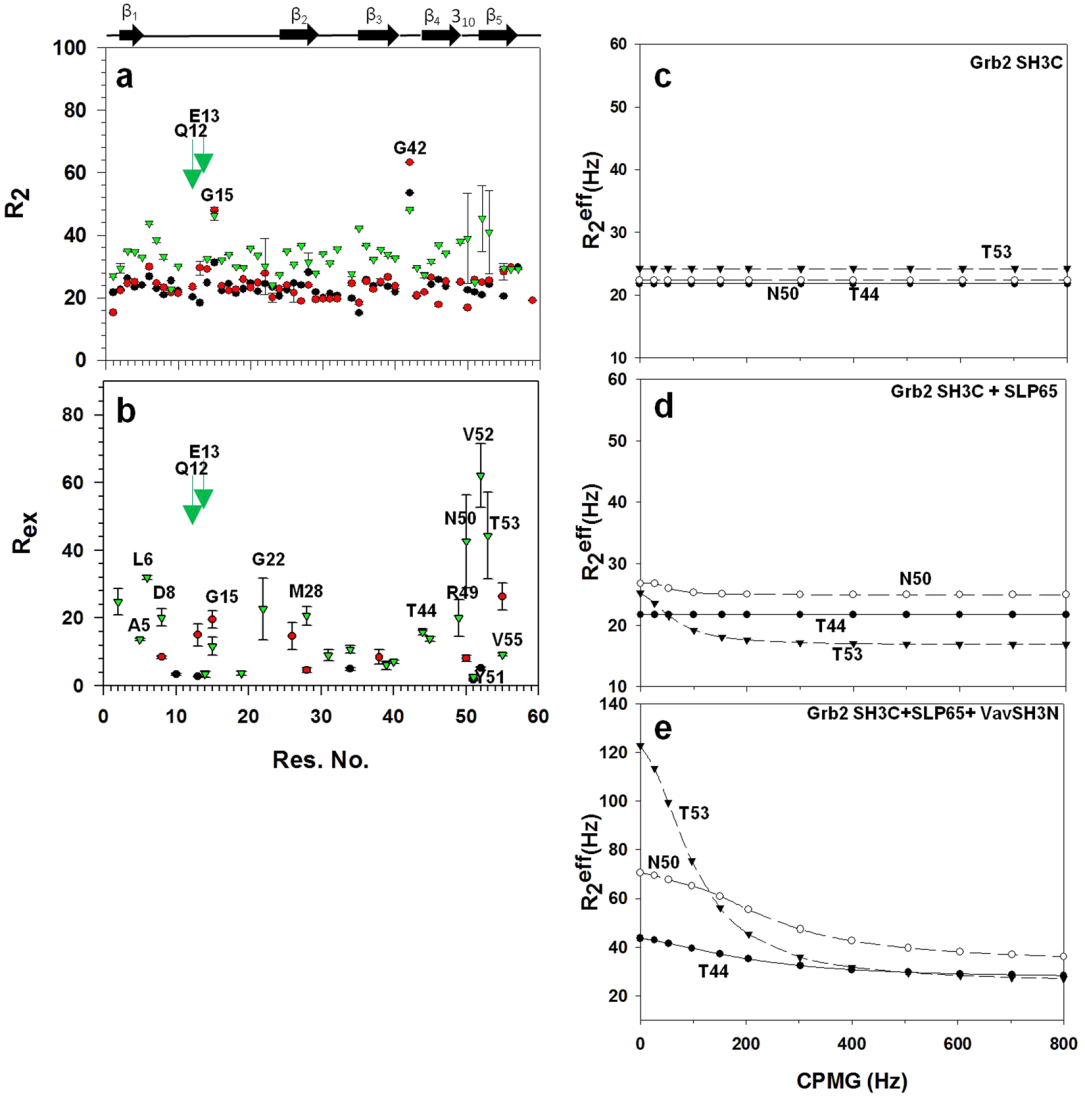
Analysis of the ^15^N relaxation dispersion experiments. (**a**) Changes in R_2_ as a function of residue number for the free Grb2 SH3C (black), Grb2 SH3C in complex with the SLP65 peptide (Complex 1, colored red), and Grb2 SH3 in complex with SLP65 peptide and Vav SH3N domain, (Complex 2, trimeric complex, colored green). R_ex_ as a function of residue number for the (**b**) free Grb2 SH3C (black), Grb2 SH3C in complex with the SLP65 peptide (Complex 1, red), and Grb2 SH3C in the SLP65-Grb2-Vav trimeric complex (Complex 2, green). ^15^N constant time relaxation dispersion profile for Thr 44, Asn 50, and Thr 53, in the (**c**) free Grb2 SH3C domain, (**d**) Grb2 SH3C domain in complex with the SLP65 peptide (Complex 1), and (**e**) Grb2 SH3C in the trimeric complex (Complex 2). The data was acquired at 298 K on a 700 MHz Bruker Avance III NMR spectrometer, equipped with a cryoprobe. The data was processed using nmrPipe^56^ and analyzed using Sparky^57^ and NESSY^62^.

In the free Grb2 SH3C, and Grb2 SH3C complexed to the SLP65 peptide, the average R_2_ values were ~24 sec^−1^, while in the trimeric complex, the values were relatively higher ~34 sec^−1^ (Fig. 9a). In the free Grb2 SH3C, Asp 10, Glu 13, Trp 34, Tyr 51 and Val 52 displayed R_2_ values greater than average, and required R_ex._ In the Grb2 SH3C-SLP65 binary complex, the following residues required R_ex_ term for data fitting to a two-state slow exchange process using Carver Richards equation^32^; Asp 8, Glu 13, Gly 15, His 26, Met 28, Gly 38, Asn 50 and Val 55 as illustrated in Fig. 8b. However, in the trimeric complex (SLP65-Grb2-Vav), a large number of residues displayed R_2_ values greater than average, and required R_ex_ term, *viz*. Tyr 2, Ala 5, Leu 6, Asp 8, Asp 14, Gly 15, Phe 19, Gly 22, Met 28, Ser 31, Asn 34, Ala 39, Ala 40, Thr 44, Gly 45, Arg 49-Thr 53, Val 55. Peaks for Gln 12 and Glu 13 in the trimeric complex could not be analyzed due to severe line broadening, and have been shown as green downwards pointing arrows in Fig. 9a,b. Most of the residues displaying conformational exchange in the SLP65-Grb2 (binary complex), and the SLP65-Grb2-Vav (trimeric complex), could be grouped together with an exchanging minor population P_B_ of ~0.3, as shown in Table 2. Individual fits of the dispersion profiles are illustrated for some of the residues in the free Grb2 SH3C domain (Fig. 9c), Grb2-SLP65 binary complex (Fig. 9d), and Grb2-SLP65-Vav trimeric complex (Fig. 9e).

**Table 2:** Physical parameters obtained by fitting the ^15^N relaxation dispersion profiles of individual residues of Grb2 in the SLP65-Grb2 binary complex as well as the SLP65-Grb2-Vav trimeric complex to a two-site exchange process using eq. 2.

	Res.	K_ex_ (s-1)	p_B_ (%)	Δδω_NCPMG_ (ppm)	Δδ_N_* (ppm)
SLP65-Grb2 (Binary complex)	Asp 8	3000 ± 316	0.08 ± 0.10	1.30 ± 0.96	0.4
	Glu 13	467 ± 505	0.30 ± 0.04	0.44 ± 0.17	0.5
	Gly 15	114 ± 69	0.30 ± 0.10	0.53 ± 0.08	1.4
	His 26	70 ± 10	0.30 ± 0.06	2.54 ± 0.70	0.0
	Met 28	22 ± 91	0.30 ± 0.08	2.46 ± 0.27	0.0
	Gly 38	146 ± 200	0.06 ± 0.10	1.42 ± 0.51	0.4
	Asn 50	438 ± 132	0.30 ± 0.09	0.30 ± 0.20	0.1
	Val 55	165 ± 146	0.30 ± 0.02	0.65 ± 0.12	0.1
SLP65-Grb2-Vav (Trimeric complex)	Tyr 2	177 ± 159	0.30 ± 0.08	1.66 ± 0.27	0.0
	Ala 5	934 ± 112	0.30 ± 0.10	0.57 ± 0.26	0.1
	Leu 6	3000 ± 215	0.13 ± 0.07	2.14 ± 0.58	0.0
	Asp 8	238	0.09 ± 0.09	1.11 ± 0.33	0.1
	Asp 14	55 ± 34	0.05 ± 0.12	1.44 ± 0.57	0.1
	Gly 15	22 ± 20	0.30 ± 0.10	4.35 ± 0.49	0.0
	Phe 19	336 ± 400	0.22 ± 0.10	0.33 ± 0.23	0.0
	Gly 22	336 ± 400	0.30 ± 0.01	0.93 ± 0.26	0.0
	Met 28	3000 ± 320	0.08 ± 0.12	2.18 ± 1.75	0.0
	Ser 31	43 ± 157	0.30 ± 0.09	1.72 ± 0.18	0.0
	Asn 34	869 ± 448	0.19 ± 0.04	3.85 ± 0.44	0.0
	Ala 39	28 ± 89	0.30 ± 0.04	0.93 ± 0.23	0.0
	Ala 40	616 ± 139	0.30 ± 0.11	0.33 ± 0.29	0.0
	Thr 44	1456 ± 325	0.30 ± 0.12	0.76 ± 0.52	0.0
	Gly 45	1794 ± 317	0.30 ± 0.12	0.78 ± 0.66	0.0
	Arg 49	1594 ± 1116	0.07 ± 0.10	1.81 ± 1.44	0.3
	Tyr 51	1442 ± 361	0.30 ± 0.02	0.3 ± 0.14	0.1
	Val 52	700 ± 439	0.30 ± 0.02	1.34 ± 0.3	0.2
	Thr 53	217 ± 352	0.30 ± 0.04	2.8 ± 1.03	0.1
	Val 55	882 ± 188	0.50 ± 0.30	0.41 ± 0.40	0.2

*Changes in the Δδ_N_ of Grb2 SH3C in the complex with Vav SH3N in a ^1^H^15^N HSQC experiment.

## Discussion

The preformed B cell receptor (BCR) transducer complex, that transports the SLP65 loaded cargo to the BCR upon stimulation, resulting in cellular activation and proliferation, comprises several highly complex and transient interactions^2, 12^. Owing to their large size, complexity, and weak nature of interaction, characterization of such complexes has remained a challenge. Hence, the mechanism of formation of several of these complexes still remains ill defined. Here, we disclose the structural organization of an interaction important for B cell function using NMR, *i.e*. the SH3 dependent trimeric complex between Grb2, SLP65 and Vav, and elucidate the molecular mechanism by which the constitutive binding of two different ligands could occur on the surface of the same Grb2 SH3C domain, in the resting B cell.

Chemical shift perturbation studies confirm that Grb2 SH3C binds the SLP65 peptide *via* its peptide binding surface, in a spatial arrangement analogous to the Mona/Gads-SLP76 peptide (PDB 1OEB)^28, 33^. The residues of the PX_3_RX_2_KP peptide crucial for this interaction are Pro_1_, Arg_5_, Lys_8_, and Pro_9_, and their mutation to an Ala has been shown to reduce/abolish binding^28^. In our NMR studies, mutation of the proline (Pro_4_) following the lysine caused a noticeable decrease in the chemical shift perturbations of Grb2 SH3C upon titration, compared to the wild type SLP65. In the crystal structure, Pro_1_ of the peptide displays weak hydrophobic stacking with Tyr 50, lysine_8_ interacts with the indole ring of Trp 35 and Arg_5_/Lys_8_ are involved in electrostatic interactions with Asp 16 and Glu 13 of Mona/Gads (Grb2 family), present in the RT loop of Grb2 SH3C. The corresponding Grb2 SH3C amides display significant chemical shift perturbations upon SLP65 binding in our studies.

Grb2 SH3C binds Vav SH3N as well, by its ligand binding surface. In the PDB structure 1GCQ, the polyproline (PP_II_) helix formed by residues 607–610 of Vav SH3N, and the following glycine, form 75% of the interaction interface, while His 634 and Asn 635 contribute to the rest^14^. Binding of the two proteins requires intact Vav, and mutation of any of the four prolines disrupts binding^15^. In the crystal structure, Asp 10/Gln 12 side chain, Glu 13 side chain, Asn 50 ND2 and Tyr 51 OH of Grb2 SH3C hydrogen bond with the side chain of Asn 635, His 634, Pro 609 carbonyl O and Pro 608 carbonyl O of Vav, respectively. These amides of Grb2 SH3C, including Asn 50 ND2, display severe line broadening upon Vav interaction in our studies, in compliance with the crystal structure.

Interestingly, SLP65 and Vav also bind Grb2 SH3C in concert, forming a trimeric complex, as suggested by several biochemical studies, as well as our own NMR chemical shift perturbation studies, relaxation dispersion measurements, and ITC measurements. The addition of a tetra proline peptide PPPPG to the Grb2-Vav complex fails to release Vav^13^. However, the SLP65 peptide “Ace-EKAPMVNRSTKPNSSS-NH2” smoothly displaced Vav SH3N from the Grb2-Vav complex, giving rise to the Grb2-SLP65 complex (Complex 1). Conversely, the addition of Vav SH3N to the Grb2-SLP65 peptide complex resulted in a trimeric complex *i.e*. SLP65-Grb2-Vav (Complex 2). Higher than average R_2_ values (transverse relaxation rate) observed for the Grb2 SH3C amides in the trimeric complex are consistent with a large τ_c_ (rotational correlation time) for the Vav bound complex. Extrapolating these results to the *in vivo* conditions in the B cell, we surmise that the Grb2-SLP65 complex must be formed prior to Vav binding.

In the Grb2-Vav binary complex (PDB 1GCQ), Pro 607 and Pro 608 of Vav SH3N interact with Phe 7, and Tyr 51 respectively, that reside in the xP binding groove 1 of Grb2 SH3C. Similarly, in the Gads-SLP76 complex, PDB 1OEB (SLP76 peptide APSIDRSTKPP which is analogous to the SLP65 peptide EKAPMVNRSTKPNSSS), Pro_2_ of SLP76 interacts with Tyr 51 of the xP binding groove 1 of Gads. To explain how the two ligands, Vav and SLP65, could be accommodated in the trimeric complex, we have proposed a model based on insights from our own ligand binding studies, as well as previous mutagenesis studies. Pro608Ala mutation of Vav SH3N displays similar affinity for Grb2 SH3C as the wild type Vav^14^, while the mutation of the corresponding proline (Pro_2_) in the SLP76 peptide diminishes its binding to Gads^28^. Pro_4_ thus seems to be a necessary requirement for SLP65 binding (analogous to SLP76) to Grb2 SH3C, unlike Pro 608 of Vav. Therefore, in our model, Pro 610 and Gly 611 of Vav bind the xP binding groove 2, lined by Trp 35 and Pro 48 (unoccupied in complex I), and Pro 607 of Vav and Pro_4_ of SLP65 bind the xP binding groove 1, interacting with Phe 7 and Tyr 51, respectively. Pro 608 of Vav SH3N most likely does not interact with the xP binding groove 1 of Grb2 SH3C in the trimeric complex. Line broadening studies suggest a slight change in the conformation/arrangement of Vav SH3N on the surface of Grb2 SH3C in the trimeric complex, compared to the Grb2-Vav binary complex. Several amides *viz*. Trp 35 (backbone and side chain) and Glu 13, in the close proximity of the xP binding groove 2 display broadening of line widths in the trimeric complex (Supplementary Fig. S3). Possibly, the polyproline helix of Vav SH3N comes closer to the xP binding groove 2. This change in conformation of Vav SH3N would move Pro 608 of Vav and Pro_1_ of SLP65 apart, (Fig. 5c), allowing the two ligands to bind Grb2 SH3C simultaneously. The change in the conformation of Vav SH3N in the trimeric complex is also reflected in the relative peak intensity change of Phe 7.

Relaxation dispersion measurements suggest an overall similarity in the dynamics of Grb2 SH3C backbone upon binding SLP65 or Vav SH3N, *i.e*. ms. time scale motions in the 3_10_ helix, and the RT loop were observed, displaying a minor population (P_B_ value) ~0.3. The conformational fluctuations were much larger upon Vav binding though. These results reflect the overlapping interaction surface of the two ligands, and their similar mechanism of interaction. The regions displaying motions in Grb2 SH3C lie close to the ligand binding surface, consistent with previous studies^34^. Relaxation dispersion experiments can detect exchange processes in the 0.3–10.0 ms time scale, arising from side chain reorientations, loop motions, secondary structure change or hinged domain movements^35^. As the residues displaying ms motions in Grb2 SH3C reside in the RT loop, and the 3_10_ helix, we speculate swinging motions/oscillations of the two secondary structures upon ligand binding.

ITC measurements suggest an overall negative free energy change upon titration with the ligands. The binding of the SLP65 peptide was enthalpically favored, while Vav SH3N binding was entropically favored, in addition to enthalpy. Entropy changes upon ligand binding can be attributed to a) changes in the desolvation entropy/hydrophobic effect (ΔS_HE_) b) loss in the conformational freedom of the protein (ΔS_RT_) and c) conformational entropy (ΔS_other_). As the ΔS_RT_ and ΔS_other_ contribute to unfavorable entropy changes, a major contribution to the total positive entropy change would come from solvation entropy^36–38^. According to the classical theory of hydrophobic effect, proposed by Frank & Evans, 1945^39^, Tanford, 1986^40^, water molecules present on the hydrophobic patches/apolar groups, are associated with unfavorable entropy component. Upon release, they gain degrees of freedom, resulting in net favorable entropy. A recent study on the SH3 domains has identified structural water molecules in several free and ligand bound structures, in conformity with the ITC results^41^. Combining our ITC data, with the insights from the crystal structure 1GCQ, and our NMR line broadening studies, we speculate that the interaction of the hydrophobic residues present in the xP binding groove 1 and 2 of Grb2 SH3C with Pro 607, Pro 610 and Gly 611 of Vav SH3N, and the concomitant release of water molecules, possibly contribute to the favorable binding entropy. Prolines, owing to their flat and rigid hydrophobic surface form van der Waals interactions with planar hydrophobic surfaces, like aromatic side chains^42^. Our ITC results are in good agreement with a previous study on Grb2 SH3C-Gab1 peptides. The G1 peptide (containing a polyproline II helix) displayed favorable entropic contribution, akin to Vav in our studies, while the G2 peptide (PPII absent) was governed by favorable enthalpy contribution and entropic penalty^43^. The entropy favored nature of Vav (containing a polyproline helix) interaction is also supported by the crystal structures of the Gab2a (2WOZ) and Gab2b (2VWF) peptide complexes of Grb2/Mona/Gads SH3C domain^44^. In Gab2a (PP_II_ present), docking was dominated by the polyproline helix, and Pro_3_ in the sequence APPPRPPKP was the first site of hydrophobic docking, while in Gab2b (PP_II_ absent), docking was dominated by electrostatic interactions of the RxxK motif^44^.

The binding affinity of free Grb2 SH3C for SLP65 and Vav SH3N was comparable in our ITC measurements. However, the difference got wider in the trimeric complex. The apparent binding-enthalpy for Vav interaction decreased, and the K_d_ became >2-fold higher in the trimeric complex. In our NMR experiments, a 6-fold Vav SH3N concentration was required to form the trimeric complex, and achieve similar line broadening, as free Grb2 SH3C. The lower affinity of Vav for Grb2 in the trimeric complex may facilitate its smooth release from the signalosome, upon interaction with lipid rafts or BCR stimulation, without perturbing the Grb2-SLP65 binary complex. Extrapolating these results to the *in vivo* conditions, where Grb2 exists as a dimer, that binds the pre-existing SLP65-CIN85 microcluster, Grb2 would bind SLP65 first, and then only Vav could associate. Notably, the trimeric complex is indispensable in the resting B cell, as a binary complex of Grb2-Vav or SLP65-Grb2 is unable to perform the same function. Grb2 and Vav, both lack the membrane localization signal, and depend on SLP65 to ferry them to the lipid rafts, by virtue of its N-terminal leucine zipper (residues 1–50). In the absence of the trimeric complex, Vav may not be localized to the membrane in the resting B cell.

Taken together, our studies unravel the mystery underlying the ligand diversity of Grb2 SH3C, and allow us to better comprehend its ligand binding site. Akin to other SH3 domains, the peptide binding site of Grb2 SH3C can be divided into three pockets, a) negatively charged specificity pocket formed by E13 and E16, that interact *via* electrostatic interactions with the basic residues of the RX_n_K motif, and b) two hydrophobic xP binding grooves, groove 1 formed by Phe 7 and Tyr 51, and groove 2 formed by Trp 35 and Pro 48, that bind a PP_II_ helix. The SLP65 peptide “EKAPMVNRSTKPNSSS”, lacks a polyproline helix (based on the SLP76 structure, 1OEB)_._ Thus, upon SLP65 binding, the xP binding groove 2, involving Trp 35 and Pro 48 of Grb2 SH3C is still unfilled, and accessible to Vav. In Gads, a relative of Grb2, the second xP binding groove of the SH3C domain is narrower and deeper compared to the Src SH3 domain, allowing an isoleucine to bind^45^. We speculate that in Grb2 SH3C as well, the xP binding grooves are deep, allowing it to accommodate a proline residue (Pro 610 of Vav), in addition to Val_6_ of SLP65 peptide. Interestingly, SLP65 is not the only Grb2 SH3C ligand lacking a polyproline helix. Several other Grb2 SH3C ligands (Group A) also lack the polyproline motif, and may similarly facilitate bivalent interactions of Grb2 SH3C, *viz*. SLP76, Gab2b, AMSH^24^, Dos, Soc, Themis-1(PPPRPPKHP)^46^ *etc*. (Table 3). A second group of Grb2 SH3C ligand (Group B) also exist, that are devoid of the RX_n_K motif *e.g*. Vav SH3N, huntingtin peptide (PPPQLPQPPP)^47^, N-WASP, and PPTPPPRP of Partner of Ral BP1 (POB1)^48^. Majority of the Grb2 SH3 ligands either fall in Group A or Group B (Table 3). A third group of Grb2 ligand have both the motifs conserved, *i.e*. a type II polyproline helix (PP_II_), and a RxxK motif, and therefore Grb2 SH3C can only bind them monovalently. These ligands include the Gab2a peptide, IQPPPVNRNLKPDRK (PDB 2VWF), HPK1 peptide, APEPGQPPLVPPRKEKMRGKMENEKRREKY, (PDB 1UTI)^33^, the tyrosine-protein phosphatase non-receptor type 23 peptide (HD-PTP) PPPRPTAPKPLL (PDB 2W10), RHYRPLPLPPLP (PDB 1IO6). Thus, Grb2 SH3C domain can bind monovalently or bivalently. The bivalent nature of ligand association justifies the need for the conservation of two different peptides of distinct composition and conformation in Gab2, as only the Gab2b peptide (PP_II_ absent) can participate in bivalent interactions^28, 44^.

**Table 3:** Peptides from different signaling proteins known to bind Grb2/Mona Gads SH3C.

Ligand	Sequence	Reference	RXXK	PP_II_	PDB
Gab1a	PDI PPP**R**PP **K**PHP	24	+	+	−
Gab1b	EPPPVD$\mathop{{\bf{R}}}\limits_{\_}$NL **K**PDR	24	+	−	−
Gab2a	APP P**R**P**P** R- **K**PSQAETPR	24	+	+	2W0Z
Gab2b	QPPP VN**R**NL **K**PDRKAKPT	24	+	−	2VWF
Ligand	PLPPLP** R** - - Y – H	-	−	+	1IO6
HPK1	PPLV PP$\mathop{{\bf{R}}}\limits_{\_}$KE **K**MRGKMENE	33	+	+	1UTI
HD-PTP	PPP RP ** T** RAP **K**PLL	33	+	+	2W10
SLP76	PAPS I D**R**ST **K**PPLDR	24	+	−	1OEB
SLP65	KAPMVN**R**ST **K**PNSSSK	27	+	−	−
c-Cbl pep1	GSQVPE**R**PP **K**PFP	24	+	−	−
AMSH	KPPVVD**R**SL **K**PGA	24	+	−	−
Dos peptide1	P P V - N**R**KL **K**P	63	+	−	−
Dos peptide2	P S V- D**R**KC **K**P	55	+	−	−
Themis	P P P - - ** R**PP- **K**HP	46	+	−	−
Soc1	P P P V D**R**SN **K**P	63	+	−	−
Cbl	PPVPP R	51	−	+	−
NoxO1	PPVVPTRPCM	55	−	+	−
N-WASP	PPPPP X R	64	−	+	−
FGFR-2	PDPMPYEPCLPQYP	65	−	+	−
Huntingtin	PPPQLPQPPP	47	−	+	−

The peptides have an RXXK motif, or a polyproline helix motif, or both. **R**XX**K** motif has been underlined and shown in bold. Superscript^R^ indicates the spatial position of the R side chain in the crystal structure.

The dual ligand binding surface of Grb2 SH3C is fairly analogous to the Amphiphysin II (Bin1) SH3C domain, that binds dynamin I (PSRPNR) and Exon 10 [(K/R)xxxxKx(K/R)(K/R)] *via* the same surface. Interestingly, both amphiphysin II CSH3 domain and Grb2 CSH3 domain also compete for the same proline sequence 338PPTPPPRP344 in POB1^48^. Bin 1 SH3C domain has numerous negatively charged residues that interact with the basic residues of Exon 10. The two ligands of Bin1 SH3C domain are mutually exclusive, giving rise to a binary switch that allows dynamin to bind only in the presence of phosphoinositides^49, 50^. Likewise, in Grb2 SH3C, SLP65 excludes Vav from the Grb2 surface, and allows it to bind only when Grb2 is complexed to SLP65. This one sided exclusion may serve twofold functions a) ensure that Vav only binds Grb2 that is associated with the SLP65 microclusters, and b) cause a marked reduction in the binding affinity of Grb2 SH3C for Vav, allowing Vav to be offloaded prior to Grb2 from the complex, upon translocating to the membrane rafts.

Notably, the trimeric complex characterized in this study is not the only Grb2 SH3C mediated multimeric complex reported till date. The constitutive association of SLP76 (atypical motif that lacks the PP_II_ helix) and Cbl (461–670 proline rich residues) with Grb2 SH3C domain in the myeloid cell line U937IF cells has also been observed. Treatment with potato acid phosphatase that disrupts phosphorylation-dependent interaction, had no effect on the Grb2-Cbl or Cbl-SLP76 interaction, suggesting that it is phosphorylation-independent^51, 52^. Similarly, the constitutive association of Grb2 SH3C with Cbl, Gab2b (lacks the polyproline helix) and LAT has also been reported in the T cell, where Grb2 SH3C interacts with the atypical PXXXR motif of Gab2b^53, 54^ and the proline rich region of Cbl, simultaneously. More recently, a trimeric complex involving NoxO1-Grb2-Cbl has been reported where the Grb2 SH3C domain binds NoxO1 peptide “PPVVPTRP”, in addition to Cbl^55^. We surmise that the bivalent mechanism of interaction may be common to several other Grb2 mediated complexes of the immune system, given the abundance of Grb2 ligands that lack the polyproline helix, but has remained elusive, partly due to its transient nature. The unique ligand binding surface of Grb2 SH3C, imparts it with the flexibility to bind a diverse range of ligands, some singly and others bivalently, to perform a biological function. In the light of the above examples and discussions, we conjecture that the peptide binding site of Grb2 SH3C needs to be redefined, and the peptide motif PX_3_RPX_2_KP expanded to include a polyproline type II helix (PP_II_) within the hydrophilic motif, to fulfill the valency of the ligand binding site of Grb2 SH3C domain.

## Methods

### Cloning, expression and purification

Mouse Grb2 SH3C domain, residues 155–217 (UniProt Q60631), Vav SH3N domain, residues 583–660 (UniProt Q8VDU4) and the proline rich region of SLP65, residues 125–330 (Q9QUN3) were cloned and expressed in *E*. *coli* by induction with 0.5 mM IPTG at 37 °C. Cells were harvested and sonicated, followed by Ni^2+^-NTA chromatography. The bound proteins were eluted with 50–200 mM imidazole. Uniformly labeled [^1^H, ^15^N, ^13^C] proteins were prepared by growing *E. coli* in M9 media, containing ^15^N NH_4_Cl (1 g/l) and ^13^C glucose (2 g/l). The N-terminal His tag was removed by thrombin cleavage using immobilized thrombin.

### NMR data acquisition

Samples used for NMR comprised of uniformly labeled [^1^H, ^15^N, ^13^C] protein, in 20 mM sodium phosphate buffer, 100 mM NaCl, pH 6.0, 0.5 mM sodium azide, 90% H_2_O, 10% D_2_O. SLP65 peptide of the sequence ‘Ace-EKAPMVNRSTKPNSSS-NH_2_’ spanning residues 201–216 was chemically synthesized.

NMR experiments were acquired on a Bruker Avance III 700 MHz spectrometer equipped with a TCI cryoprobe, and a Varian Inova 500 MHz NMR spectrometer with HCN probe, both the instruments installed at the National Institute of Immunology, New Delhi. NMR data processing was carried out on a workstation running Red Hat Enterprise Linux 5.0, using NMRPipe/NMRDraw^56^ and analyzed using Sparky^57^. Experiments were performed at 298 K. The data were multiplied by a phase shifted sinebell apodization function in all dimensions.

^1^H^15^N HSQC spectra were acquired using 1024 data points (t2) dimension and 512 data points (t1) dimension. CBCAcoNH, HNCACB and HNcoCA experiments were collected with 1024 (t3) × 48 (t1) × 24 points (t2). ^1^H TOCSY experiments were acquired with 1024 points in the (t2) dimension and 512 points in the (t1) dimension. Data was linear predicted in the forward direction for up to half the number of experimental points in the indirect dimension. ^15^N ^13^C Spectra were referenced indirectly using Sodium 2, 2-dimethyl-2-silapentane-5-sulfonate (DSS) as standard^58^. For temperature measurements, methanol was used as an external standard^59^.

### Chemical shift perturbations

Changes in HN have been reported as average chemical shifts (ΔΔδ_HN_) derived from the Equation 1 ^60^

1 $${{\rm{\Delta }}{\rm{\Delta }}{\rm{\delta }}}_{{\rm{HN}}}={[{({{\rm{\Delta }}}_{{\rm{HN}}})}^{2}+{({\rm{\Delta }}{\rm{N}}/5)}^{2}]}^{1/2}$$

where Δ_HN_ is the change in the chemical shift in the proton dimension, and ΔN is the change in the chemical shift in the nitrogen dimension.

### Relaxation Dispersion experiments

Constant-time ^15^N CPMG relaxation dispersion experiments were acquired at 298 K on a 700 MHz NMR spectrometer equipped with a cryoprobe using CPMG pulse sequences as described^61^. A T_relax_ value of 40 ms was used. A reference spectrum was acquired, without a constant-time CPMG element, along with 10 spectra with varying CPMG frequencies *viz*. 25, 50, 100, 150, 200, 250, 300, 400, 600 and 800 Hz in duplicates. Sixteen scans per FID were recorded with a relaxation delay of 2.5 s. The pseudo-3D data were processed using nmrPipe^56^ and peak intensities were measured using Sparky^57^.

### Relaxation data analysis

R_2_^eff^ was extracted from a series of CPMG constant-time relaxation dispersion experiments using NESSY^62^, according to the Equation 2:

2 $${{\rm{R}}}_{2}^{{\rm{eff}}}=1/{{\rm{T}}}_{CPMG}\,\mathrm{ln}\,{\rm{I}}(0)/{\rm{I}}({v}_{CPMG})$$

where T_*CPMG*_ is the constant CPMG time, I(0) represents the peak intensity in the reference spectrum while I(ν_*CPMG*_) is the intensity of the peak at the CPMG frequency (ν_*CPMG*_). Dispersion profiles of the residues were individually fitted to different models that are distinguished from one another as follows:

- Model 1 with no exchange, *i.e*.
  3 $$\,{{\rm{R}}}_{2}^{{eff}}={{\rm{R}}}_{2}^{0}.$$
- Model 2 with two site fast exchange
  4 $${{\rm{R}}}_{2}^{eff}={{\rm{R}}}_{2}^{0}+{\rm{\varphi }}/{{\rm{k}}}_{{\rm{ex}}}[1-{\underline{4v}}_{\mathop{\underline{CPMG}}\limits_{{{\rm{k}}}_{{\rm{ex}}}}}\,\tanh ({{\rm{k}}}_{{\rm{ex}}}/4{v}_{CPMG})]$$
  where ϕ = *p*_a_ *p*_b_δw^2^.
- Model 3 with two site slow exchange using automated fitting software NESSY^62^

5 $${{\rm{R}}}_{2}^{eff}={{\rm{R}}}_{2}^{0}+{{\rm{k}}}_{{\rm{ex}}}/2-{v}_{CPMG}\,cos{h}^{-1}[{D}_{+}cosh({\eta }_{+})-{D}_{-}cos({\eta }_{-})]$$

where ν_*CPMG*_ = (τ_cp_ + *p*ω_180_°)/2

$$\begin{matrix}{{\rm{D}}}_{\pm } & = & 1/2[\pm 1+({\rm{\Psi }}+2{{\rm{\delta }}{\rm{\omega }}}^{2})/{({{\rm{\Psi }}}^{2}+{{\rm{\xi }}}^{2})}^{1/2}]\\ {{\rm{\eta }}}_{\pm } & = & {[\pm {\rm{\Psi }}+{({{\rm{\Psi }}}^{2}+{{\rm{\xi }}}^{2})}^{1/2}]}^{1/2}/(2\sqrt{2{\nu }_{CPMG}})\\ {\rm{\Psi }} & = & {{{\rm{k}}}_{{\rm{ex}}}}^{2}-{{\rm{\delta }}{\rm{\omega }}}^{2}\\ {\rm{\xi }} & = & -2{\rm{\delta }}{\rm{\omega }}({p}_{a}{{\rm{k}}}_{{\rm{ex}}}-{p}_{b}{{\rm{k}}}_{{\rm{ex}}}).\end{matrix}$$

In the above equations, ν_*CPMG*_ is the field strength of the CPMG spin-echo pulses, τ_cp_ is the delay between two consecutive 180° ^15^N refocusing pulses, *p*ω_180_° is the pulse width, R_2_° is the transverse relaxation rate constant in the absence of exchange, *p*_a_ and *p*_b_ are the equilibrium populations at the two sites, k_ex_ is the exchange rate constant, and δω is the chemical shift difference between the two sites, respectively.

From the above analysis, the following parameters were obtained a) the rates of inter-conversion of various conformations k_ex_, b) relative populations of the exchanging species *p*_*b*_ c) difference in chemical shifts between the two species Δδω_N_ and d) χ^2^_res_ for individual fits.

### Isothermal Titration Calorimetry measurements (ITC)

ITC measurements were carried out on a VP-isothermal titration calorimeter, MicroCal, Inc (USA). The proteins were dialyzed for 24 hours against 20 mM Sodium Phosphate buffer, 100 mM sodium chloride, pH 6.0, followed by degassing. The SLP65 peptide solution was also prepared in the same buffer. All titration experiments were carried out at 25 °C using a syringe, constantly stirring the sample. In case of SLP65 peptide titration, stirring was carried out at 300 rpm, while the Vav SH3N titration was done at 200 rpm. For the binding isotherms, the protein solutions (20 μM of Grb2 SH3C for the peptide titration and 40 μM for the Vav titration) were titrated with 18–28 injections of the titrant (10ul of 600 μM Vav, and 10ul of 280 μM SLP65 peptide). Each injection was of 5 sec duration, followed by 4.5 min interval. The enthalpy change (ΔH) and binding constant (K_a_) were obtained directly using Origin software, Version 7.0 (MicroCal, U.S.A). Other thermodynamic parameters were calculated using the formula ΔG° = RT*ln*K_d_.

## Electronic supplementary material

Supplementary Fig. S1, S2 and S3
