# Supplementary material for: Grb2 carboxyl-terminal SH3 domain can bivalently associate with two ligands, in an SH3 dependent manner
Source: Sci Rep. 2017 Apr 28;7:1284. doi: 10.1038/s41598-017-01364-5 (PMC5430726; doi:10.1038/s41598-017-01364-5)
Supplement: Supplementary file 1 — Supplementary Fig. S1, S2 and S3 [file 41598_2017_1364_MOESM1_ESM.pdf]

Grb2 carboxyl-terminal SH3 domain can bivalently associate with two ligands, in an SH3 dependent manner

Richa Arya[†], Rohit Singh Dangi[‡], Pinakin K. Makwana[‡], Ambrish Kumar[‡], Santosh Kumar Upadhyay[¶] and Monica Sundd[‡]*

[‡]National Institute of Immunology, Aruna Asaf Ali Marg, New Delhi 110 067, India.

[†]Department of Biochemistry, University of Delhi South Campus, Benito Juarez Road, New Delhi 110 021, India

[¶]CSIR-Institute of Genomic and Integrative Biology, Mathura Road, New Delhi-110 025

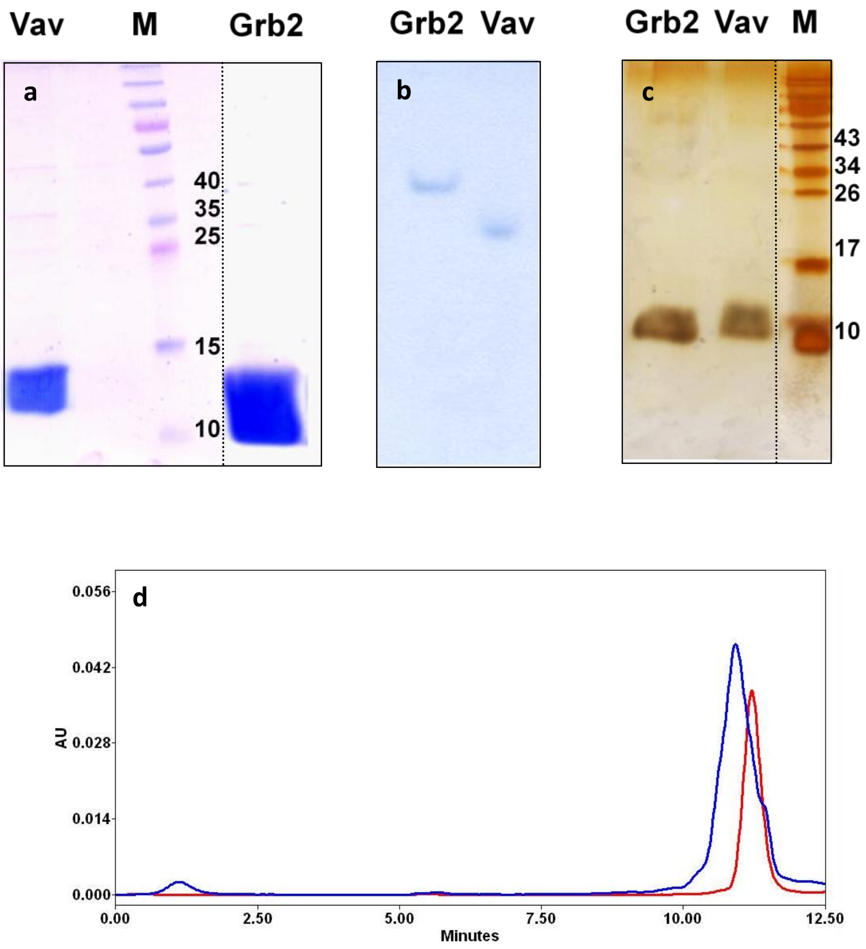

Supplementary Fig. S1. **Assessing purity of the protein samples.** Coomassie stained a) SDS-PAGE, b) Native-PAGE, c) Silver stained SDS-PAGE with samples cross linked using 0.05% glutaraldehyde, after incubation (100µg protein in 20µl final volume) for 20 min. at 37°C. Lane from a different gel/part of the gel is marked as a dotted line. d) Size exclusion chromatography of His-tag cleaved Grb2 SH3C domain (blue) and Vav SH3N domain (red).

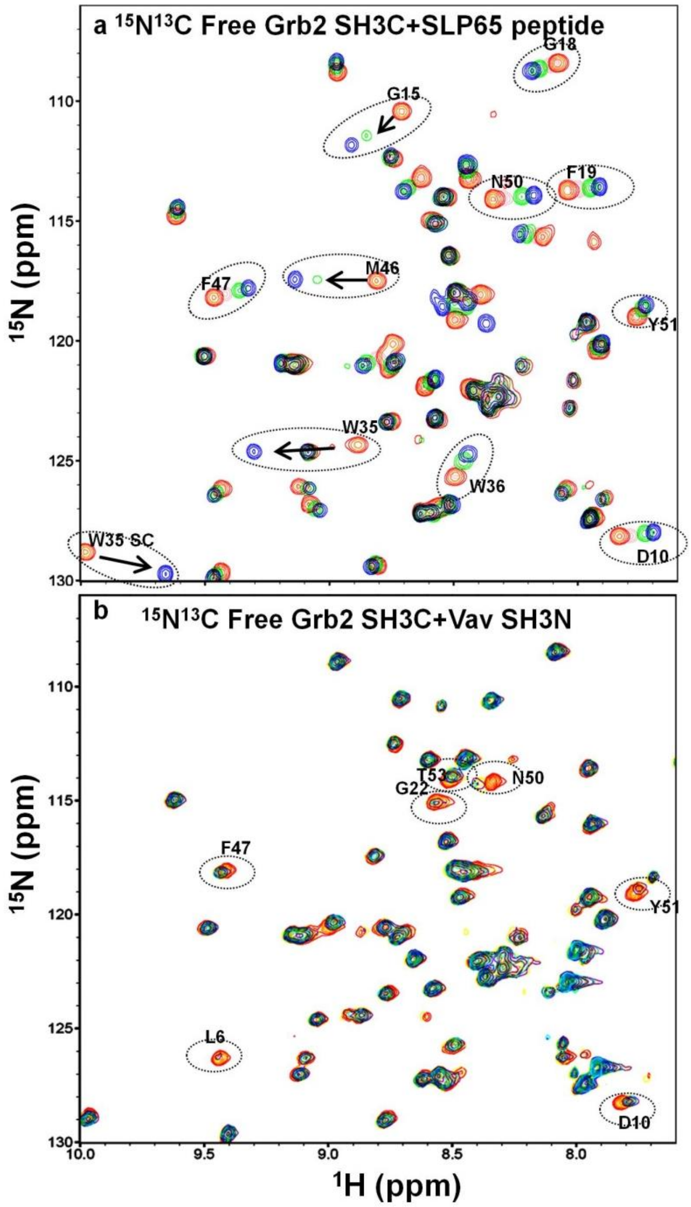

Supplementary Fig. S2. **$^{1}H^{15}N$ HSQC spectra of Grb2 SH3C domain upon titration with its ligands.** (a) $^{1}H^{15}N$ HSQC spectra of free Grb2 SH3C (red) superimposed on its spectra in presence of 0.4 (pink), 1.4 (green) and 2.0 (blue) fold molar excess of SLP65 peptide acquired on a 500 MHz Varian Innova NMR Spectrometer. (b) Multiple overlaid spectra of free Grb2 SH3C (colored red) and spectra containing 0.4 (yellow), 1.0 (maroon), and 2.0 fold molar excess of Vav SH3N (cyan). The spectra were acquired on a 700 MHz Avance III Bruker NMR spectrometer.

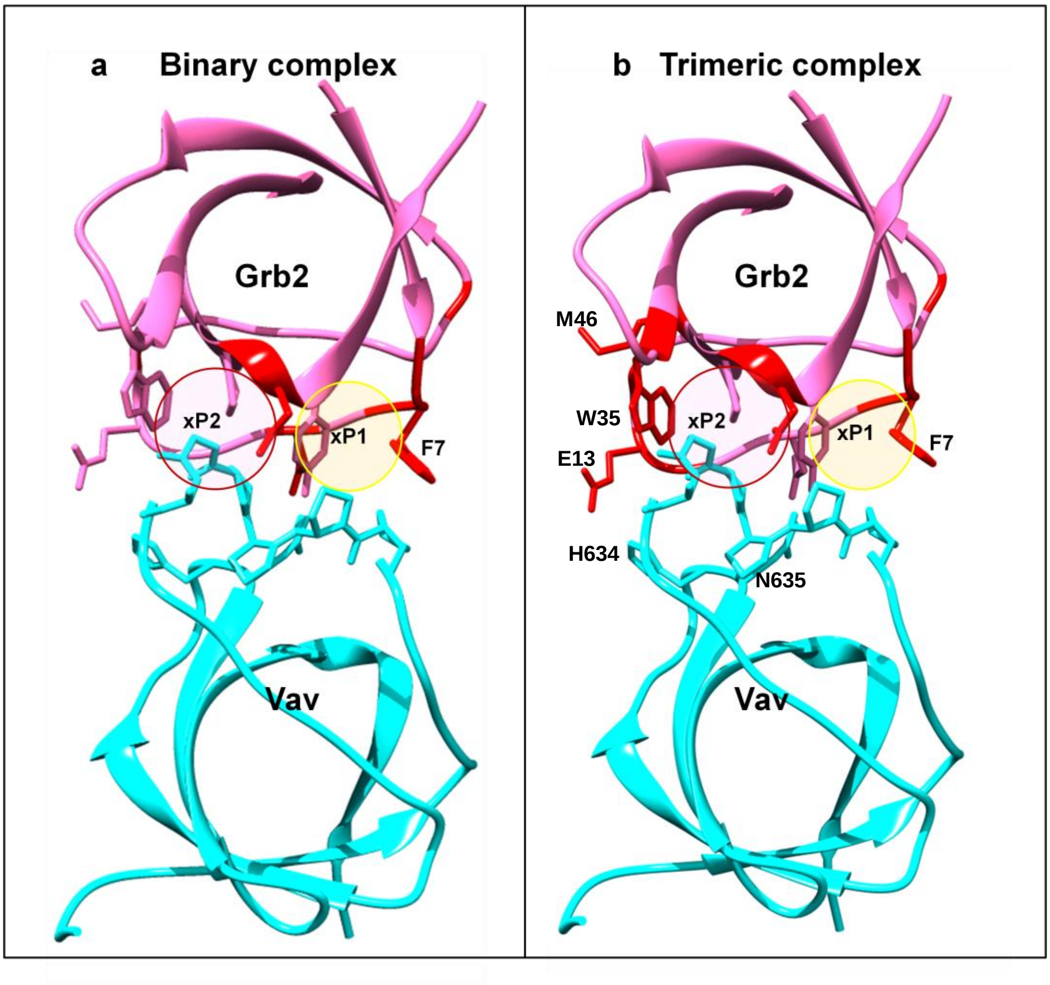

Supplementary Fig. S3. **Grb2 SH3C amides displaying line broadening upon Vav SH3N binding.** Broadening of line widths observed in the $^{1}H^{15}N$ HSQC spectra of Grb2 SH3C upon binding Vav SH3N, have been mapped to the a) binary complex, and b) the trimeric complex (Complex 2), based on the PDB ID 1GCQ . Residues displaying line broadening are colored red.
